# A Novel Strategy for Enhance Potentiation of Meglumine antimoniate against *Leishmania major* In Vitro

**Published:** 2019

**Authors:** Farzaneh MIRZAEI, Hossein KHANAHMAD, Fatemeh NAMDAR, Shahrokh IZADI, Seyed Hossein HEJAZI

**Affiliations:** 1. Department of Parasitology and Mycology, School of Medicine, Isfahan University of Medical Sciences, Isfahan, Iran; 2. Department of Genetics and Molecular Biology, School of Medicine, Isfahan University of Medical Sciences, Isfahan, Iran; 3. Department of Medical Parasitology and Mycology, School of Public Health, Tehran University of Medical Sciences, Tehran, Iran; 4. Skin Diseases and Leishmaniasis Research Center, Department of Parasitology and Mycology, School of Medicine, Isfahan University of Medical Sciences, Isfahan, Iran

**Keywords:** Leishmaniasis, *L. major*, Meglumine antimoniate, In vitro, Listeriolysin O

## Abstract

**Background:**

We aimed to design a different method of drug delivery for increased transfer of the choice drug (meglumine antimoniate) within the host cells. Therefore, listeriolysin O (LLO), a bacterial product which is a member of pore-forming peptides was used as an enhancer factor with meglumine antimoniate in order to facilitate the transition of the drug across macrophage membrane.

**Methods:**

LLO was produced in Isfahan University of Medical Sciences in 2016, by expressing the *hlyA* gene in *Escherichia coli* and purified using affinity chromatography. Cytotoxicity of the purified protein was investigated in an in vitro model of macrophage *Leishmania* infection.

**Results:**

LLO was cytotoxic against murine macrophage cells (J774-A1) and amastigote forms of *L. major* (MRHO/IR/75/ER). It was less toxic to macrophages (CC50=2.56 μg ml^−1^ ±0.09) than to the parasites (IC50=1.72 μg ml^−1^ ±0.07). Moreover, noncytotoxic concentration of LLO (0.006 ug ml^−1^) potentiated the cytotoxicity induced by low dose concentration of meglumine antimoniate. Very little dose of meglumine antimoniate was needed when combined with the LLO (IC50=12.63 μg ml^−1^ ±0.13) in comparison with the cytotoxicity induced when the drug is used alone (IC50=46.17 μg ml^−1^ ±0.28).

**Conclusion:**

The combination of pore-forming proteins with anti-leishmanial agents could increase the advantage of anti-leishmanial drugs. Since lower concentrations of anti-leishmanial drugs can reduce undesirable side effects of chemotherapy trials carried out in animal models and then in humans with this system.

## Introduction

Leishmaniasis is an infectious disease caused by obligate intracellular parasitic protozoa of the genus *Leishmania* in the family Trypanosomatidae (1). It is including 20 species that are pathogenic for humans and affecting more than 12 million people in 98 countries with up to 350 million people are at risk of infection (2).

Clinical manifestations mainly including cutaneous (CL), mucocutaneous (MCL) and visceral (VL) forms (3). CL is the most common and demonstrate more than 50% of new cases reported (4) and presents in two main forms: Anthroponotic cutaneous leishmaniasis (ACL) and zoonotic cutaneous leishmaniasis (ZCL), caused by *L. tropica* and *L. major*, respectively (5). It is endemic in many parts of Iran with a high incidence rate (6). The parasite has two morphological forms in life cycle: The promastigote in the digestive organs of the vector, female phlebotomine sand fly, and the amastigote in the macrophages (and rarely other cell types) in the mammalian host (7, 8).

Pentavalent antimony [Sb(V)] containing drugs such as meglumine antimoniate (MA) and sodium stibogluconate (Pentostam) is the first choice for the treatment of CL (9). Since there is no effective vaccine for prevention against leishmaniasis, rapid diagnosis and appropriate chemotherapy, appear to be the only ways to control of the disease, but these drugs have several disadvantages including high toxicity and many undesirable side effects (thrombocytopenia, pancreatitis and cardiac arrhythmia), high cost, drug resistance and must be administered in repeated injections with painful (10).

Furthermore, in recent years, the resistance of *Leishmania* spp. to MA has reported by a number of researchers and it is a major clinical impediment in endemic areas such as Iran (11–13). Hence during recent decades, many efforts have been made to increase effective new compounds for treatment of CL that would be to develop novel drug delivery systems (DDS) such as liposomes, polymeric nanoparticles, and solid lipid nanoparticles in order to improve the efficacy, tolerability and lower drug toxicity (14). Another method used to improve drug delivery mechanism is the use of pore-forming peptides (15, 16). These peptides can generate a pathway for increased penetration of therapeutic agents across cellular membranes by creating pores in the cell wall (17).

LLO is a member of pore-forming peptides, belonging to the family of cholesterol-dependent cytolysins (CDCs), with optimum hemolytic activity at acidic pH (around pH 5–5.5) and produced by different bacterial species including *Listeria monocytogenes* (18). This exotoxin, encoded by the *hlyA* gene, composed of 529 amino acids with a predicted molecular weight of 58 kDa. Several monomer subunits of LLO bind to cholesterol-containing cell membranes and oligomerize to form transmembrane pores of approximately 20 nm in diameter (19). These pores are particularly large and allow the transmission of macromolecules such as peptides and genes (18).

Therefore in this study, we used a novel strategy, the use of LLO in a low concentration to increase the therapeutic efficacy of meglumine antimoniate and its facilitating access into the infected cells to improve DDS for treatment of CL.

## Materials and Methods

### Bacterial strains, kits and reagents

BL21 (DE3) and DH5α strains of *Escherichia coli* (Novagen, Madison, WI, USA) were used for cloning and expression steps. Agarose gel DNA extraction kit, plasmid extraction kit, chemical agents for SDS-PAGE, western blotting and Ni-NTA agarose resin were purchased from Qiagen (Valencia, CA, USA).

### Construct Design and Cloning of LLO

Amino acid sequence of the *hly A* (Gene Bank Accession No. M24199) was back-translated to nucleotide sequence. Codon optimization for enhanced *E. coli* expression was performed by JCAT server at http://www.jcat.de. The optimized gene fragment with *Nco1* and *Xho1* sites was synthesized by GenCust Europe (Dudelnag, Luxembourg) and subsequently cloned into pET-28a to construct pET-LLO plasmid.

### Expression and purification of the recombinant protein

To this purpose, we have used the method (18) with some modifications in Isfahan University of Medical Sciences in 2016. Briefly, the pET-28a recombinant vector was transformed into *E. coli* BL21 (DE3) strain by CaCl2 method. The transformed clones were selected on LB (Luria-Bertani) agar plates containing 30 μg ml^−1^ kanamycin. Several of the selected colonies were cultured in 5 ml of LB medium and the culture was grown at 37 °C, expression was induced by the addition of 1mM IPTG (Isopropyl-b-D-thioga lactopyranoside) and analyzed by SDS-PAGE (12 %). The colony with the most high expression of the recombinant protein was selected and cultured in 200 ml of LB medium containing 30 μg ml^−1^ kanamycin. The culture was grown at 180 rpm and 37 °C to an optical density at 600 nm (OD 600) of 0.5–0.7. The recombinant protein expression was induced by adding 1 mM IPTG to the medium and incubated at 22 °C for 16 h. The cells were harvested 4 h after induction by centrifugation at 7000 rpm/10min at 4 °C and the pellet was resuspended in 20 ml of lysis buffer (50 mM NaH2PO4 (pH 7.3), 300 mM Nacl, 2mM Dithiothreitol (DTT), 10 mM Imidazole, 5% (v v^−1^) Glycerol). The cells were incubated on ice and sonicated for three min in 15 s bursts. Then, the lysate was centrifuged at 14,000 rpm for 30 min at 4 °C to remove cell debris. The supernatants were applied to a chromatographic column (10 ml) containing Ni–NTA agarose resin and incubated at 25 °C for 1 h, equilibrated with the native wash buffer (50 mM NaH2PO4, 300 mM NaCl and 20 mM Imidazole, pH 8.0). Unbound proteins were eluted with the above buffer and the bound LLO was eluted by the same as wash buffer except for Imidazole 300 mM. The presence of the protein and the purity of the eluted fractions were analyzed by 12% SDS-PAGE and Coomassie brilliant blue (R=250) staining. Then, fractions contained the LLO Protein were dialyzed against a dialysis buffer (Tris buffer 100 mM pH=8.0, NaCl 25 mM, 2ME 5 mM). The Protein concentration was determined by the Bradford protein assay using bovine serum albumin as the standard.

### Western Blot Analysis

The recombinant protein was detected by western blot analysis. Electrophoresed proteins (SDS-Page) were transferred to a nitro-cellulose membrane using a semi-dry method in a transfer buffer (25 mM Tris, 192 mM glycine, 20% methanol) at 90 mA for 60 min. The membrane was then blocked overnight at 4 °C in 5% skimmed milk in Tris-buffered saline buffer (TBS), pH 7.6. Then, the membrane was incubated in a 1:1,000 dilution of anti-histidine horseradish peroxidase antibody with gentle shaking for 1 h at room temperature. The membrane was washed with PBST (phosphate buffered saline/Tween 20) three times after each incubation period. Visualization was performed using TMB stabilized substrate for HRP (Promega Corporation, Madison, USA) (20).

### Parasite and Cell Culture

*L. major* promastigotes (MRHO/IR/75/ER) were kept frozen, provided from the Department of Parasitology of Isfahan University of Medical Science, Isfahan, Iran. The parasites were cultured in RPMI 1640 supplemented with penicillin (100 IU ml^−1^), streptomycin (100 μg ml^−1^) and 10% heat-inactivated fetal calf serum (FCS), at temperature of 24 °C. Murine macrophage cell line, J774-A, were purchased from Pasteur Institute of Iran (Tehran, Iran). The cells cultured in RPMI-1640 supplemented with 2 mM L-glutamine, 10% FCS, 100 μg ml^−1^ streptomycin, 100 IU ml^−1^ penicillin at 37 °C and 5% CO2 (21).

### Cytotoxicity assay

In vitro cytotoxicity of the purified LLO preparation was tested on J774-A1 cells. The macrophages suspension in free-serum RPMI 1640 medium (1×10^6^ ml^−1^) of 20 μl, was mixed with 30 μl of LLO preparation diluted with buffer A solution (10 mM MES pH 5.5 containing 140 mM NaCl, 1 mM EDTA with 2 mM DTT) at various concentrations (concentration range 0.006–213 ug ml^−1^), and immediately incubated for 30 min at 37 °C. Macrophages suspensions in a culture medium with Amphotericin B and without drug were used as positive and negative controls, respectively. Cell mortality was determined by trypan blue exclusion assay. Mixing cells with trypan blue solution (0.4% v v^−1^) and dead versus total cells were manually counted in a hemocytometer and an optical microscope. Cell mortality was calculated as the percentage of dead cells compared to the total number of counted cells (22). The 50% cytotoxic concentration (CC50) was determined by regression analysis using the SigmaPlot™13 software. Each assay carried out in triplicate in three independent experiments.

### Leishmania-Macrophage Infection Model

Anti amastigote activity of LLO preparation was performed using the methods (23). Briefly, at first 2 cm^2^ coverslips placed in the wells of 6-chamber slides (Lab-Tek, Nalge Nunc International, NY, USA). In the next step, 200 uL of macrophage cells suspension (J774-A1, 10^5^ mL^−1^) added in each well and incubated at 37 °C in 5% CO2 for 24 h. Then, 200 uL (10^6^ mL^−1^) promastigotes in stationary phase added into it (10:1) and incubated again at the same condition for 24 h. Free parasites removed by washing the wells with RPMI-1640 medium.

### In vitro amastigote Assay

Infected macrophages treated with 50 uL of various concentrations (0.006–213 ug mL^−1^) of the LLO (diluted with buffer A) or MA alone and also various concentrations of MA (0.006–213 ug mL^−1^) along with 0.006 ug mL^−1^ of LLO diluted with buffer A at 37 °C in 5% CO2 for 48 h. Finally, the dried slides fixed with methanol, stained by Giemsa, and studied under a light microscope. Furthermore, infected macrophages with no drugs and non-infected macrophages with no drugs were considered as positive and negative controls, respectively. Anti-leishmanial effects were measured by counting the number of amastigotes in each macrophage by examining 100 macrophages on each coverslip and comparing them with those obtained in positive control. IC50 was calculated using SigmaPlot™13 (Systat Software Inc, USA). Each experiment performed triplicate.

### Statistical analysis

The results are presented as the mean±SEM of independent experiments. The one-way analysis of variance statistical (ANOVA) test was used to assess the significance of the differences among the various groups. In the case of a significant *P-*value, the multiple-comparison Tukey test was used to compare the means of the different treatment groups. *P*-values≤0.05 was considered as statistically significant. Data analysis was carried out using software SPSS 19.0 (IBM, Chicago, IL, USA). The IC50 calculated and the graphs plotted using SigmaPlot™13 (Systat Software Inc, USA).

## Results

### Expression, Purification, and Protein Analysis

**Fig. 1:**
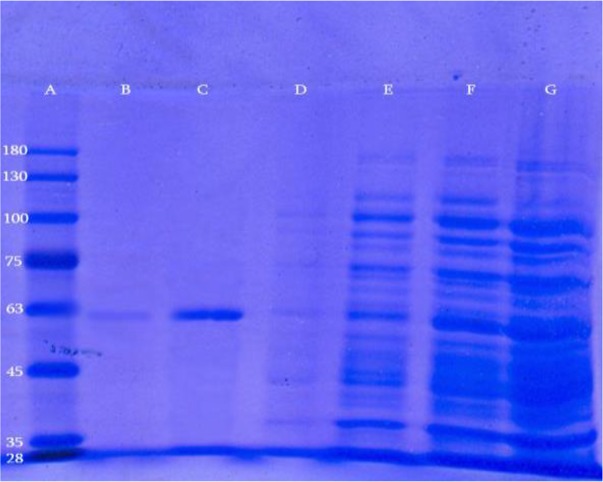
Purification of recombinant LLO protein with 6X-His-tagged from pET28a by Ni-NTA affinity column. Lanes A-G indicate protein molecular weight marker, second eluted fraction, first eluted fraction, wash column, flow-through of the column, soluble soup before running on the column, Pellet, respectively.

The synthetic *hly* gene was successfully cloned in *E. coli* BL21 (DE3) cells by using pET-28a as an expression vector. The recombinant His-tagged LLO was successfully purified by applying Ni-NTA affinity column chromatography under denaturing condition and analyzed by SDS-PAGE. This analysis represented a single major band with a molecular mass of about 58 kDa as shown in Fig. 1.

**Fig. 2:**
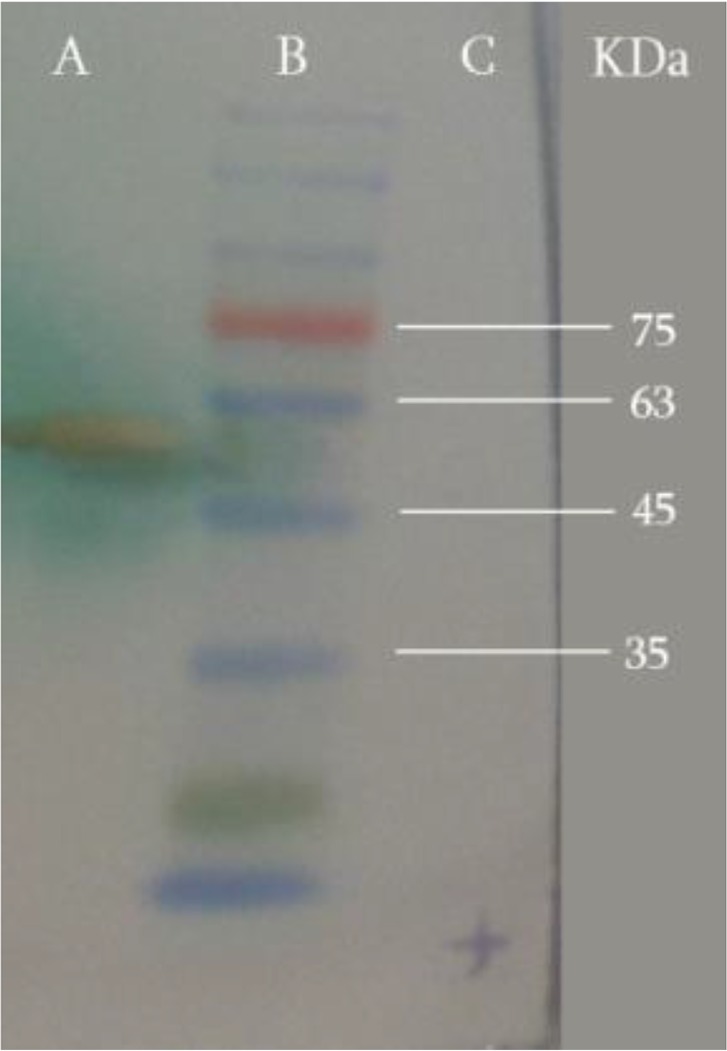
Western blot analysis with HPR-labeled monoclonal anti-His-tag antibody. Lane A: The purified LLO, shows one band at 58 kDa. Lane B: prestained protein marker, Lane C: total protein before induction

Western blotting technique with HRP-labeled monoclonal anti-His-tag antibody confirmed the identity of expressed recombinant protein at 58 kDa (Fig. 2). The concentration of purified LLO measured by Bradford protein assay was about 0.856 mg per 200 ml of bacterial culture.

### Note

#### Cytotoxicity assay

The results of assessment of *in vitro* cytotoxic activity of the purified LLO preparation on J774-A1 cells are shown in Fig. 3. The value of 50% cytotoxic concentration (CC50) of this protein on macrophages was 2.56 μg ml^−1^ ± 0.09. In addition, LLO did not exert cytotoxicity under 0.012 μg ml^−1^ (Fig. 3a).

**Fig. 3:**
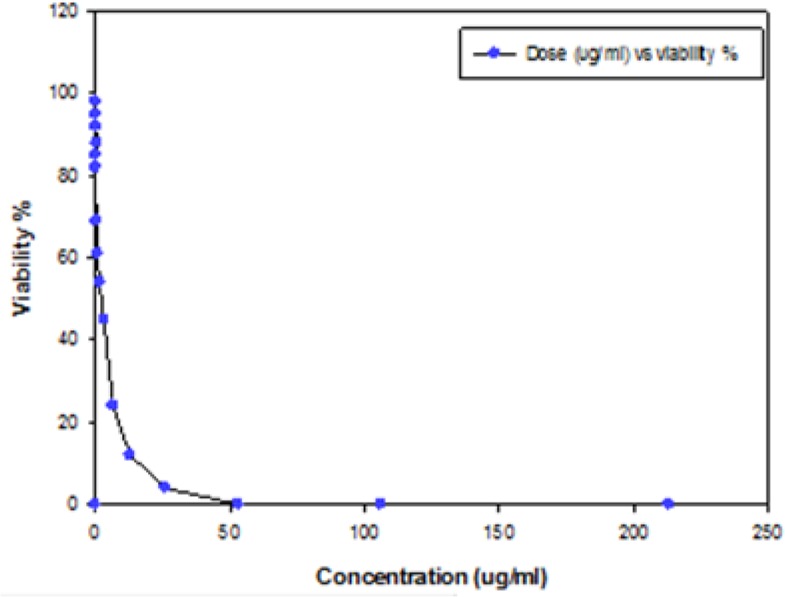
The calculation of CC50 of LLO, using the results of different concentration of LLO on macrophage (J774-A1) at 16 concentrations in serial dilution starting from 0.006 ug ml^−1^. the percentage of cellular viability, determined by Trypan Blue exclusion assay after treatment with the indicated doses of LLO after 30 min at 37°C

**Fig. 3a:**
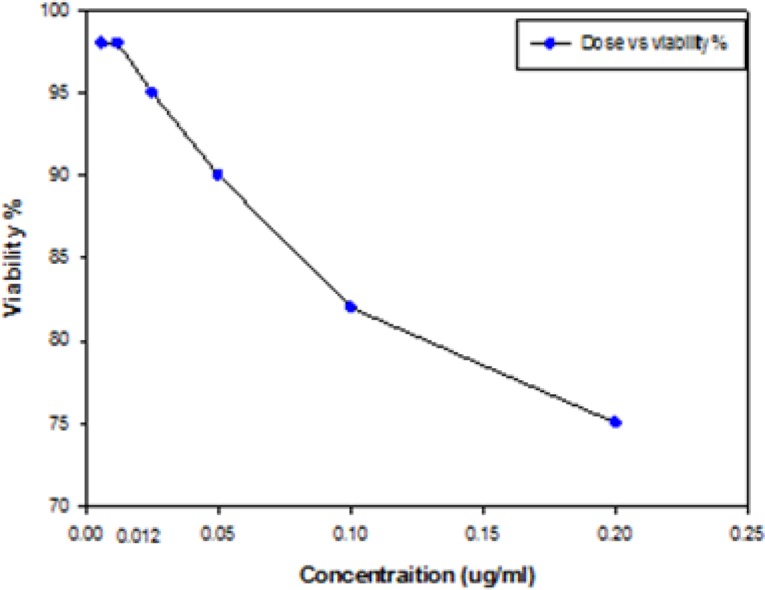
Graph shows the percentage of cellular viability, determined by Trypan Blue exclusion assay after treatment with the indicated doses of LLO after 30 min at 37°C

### In vitro amastigote Assay

The in vitro anti-amastigote effects of the purified LLO preparation were examined by measuring the mean number of amastigotes in each infected macrophage.

**Fig. 4:**
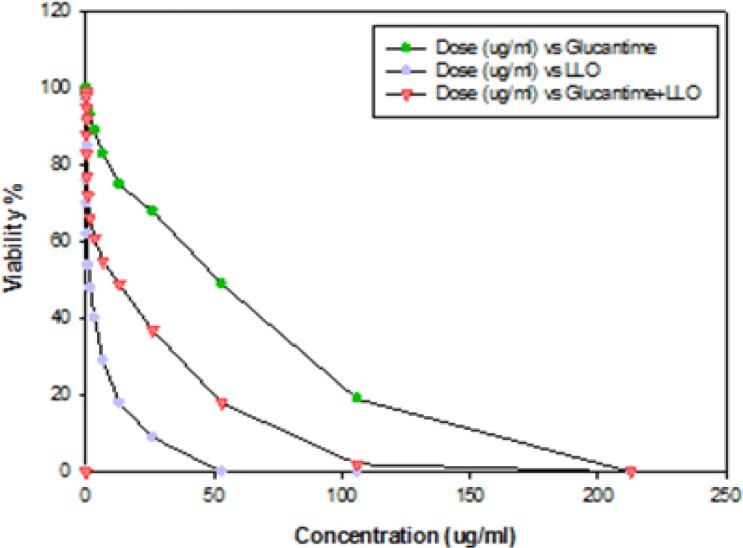
The calculation of IC50 of Meglumine antimoniate (MA) (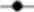) and LLO (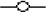) using the results of different concentration of MA or LLO on amastigote forms of L. major at 16 concentrations in serial dilution starting from 0.006 ug/ml. Moreover, for calculation of IC50 of MA+ LLO (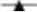), using the results of different concentration of MA (0.006–213 ug ml^−1^) along with noncytotoxic concentration of LLO (0.006 ug ml^−1^) on amastigote forms of L. major. The percentage of cellular viability, measured by counting the number of amastigotes in each macrophage by examining 100 macrophages on each coverslip and comparing them with those obtained in positive control with light microscope after 48 h incubation at 37°C, 5% CO2

**Fig. 5:**
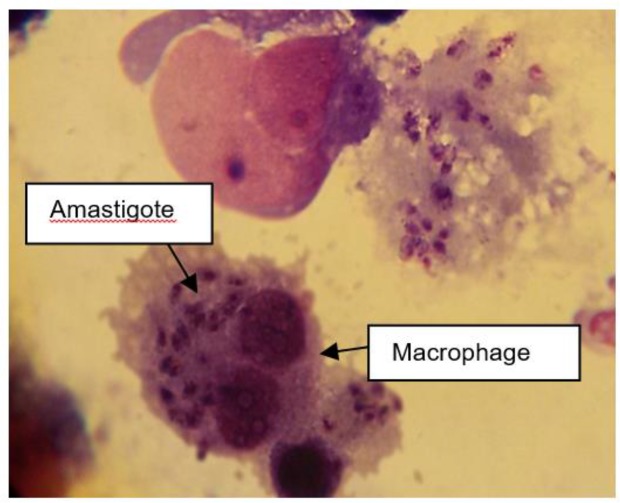
Infected macrophages with no drugs (positive control).

**Fig. 6:**
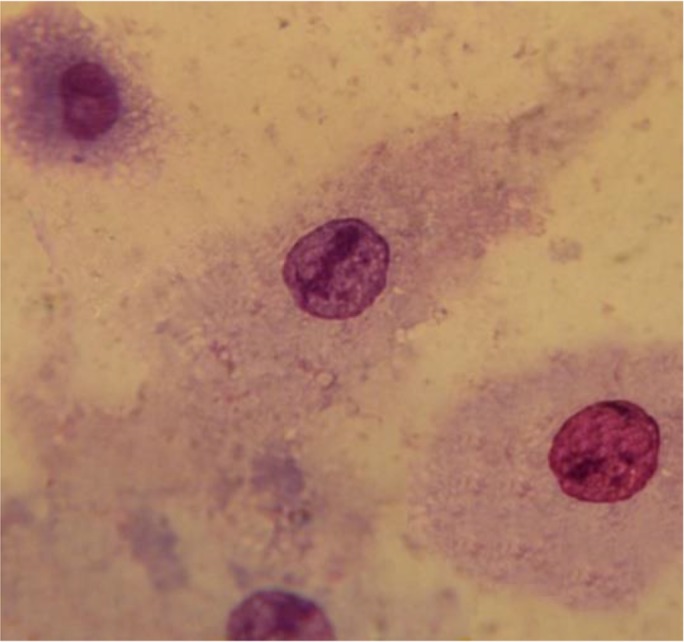
the effect of MA plus LLO at high concentration of MA on amastigote forms of L. major after 48 h incubation at 37°C, 5% CO2.

**Fig. 7:**
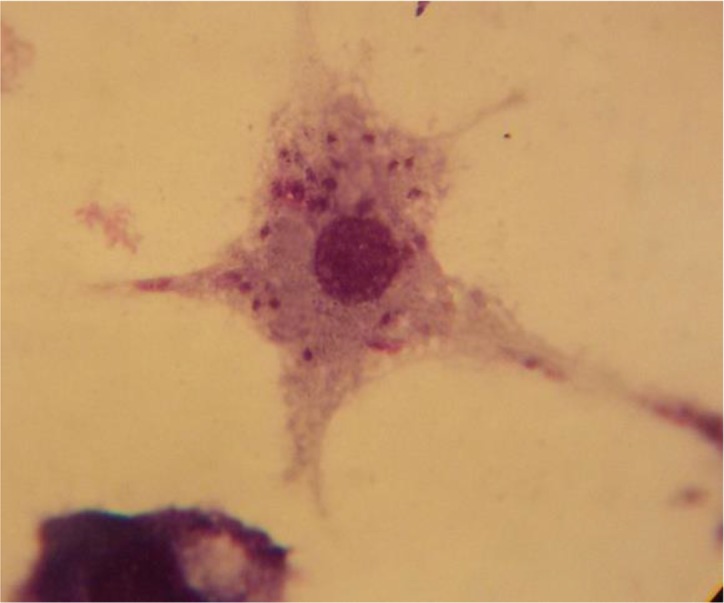
The effect of MA at a concentration of 12.63 ug ml-1 alone on amastigote forms of L. major after 48 h incubation at 37°C, 5% CO2.

**Fig. 8:**
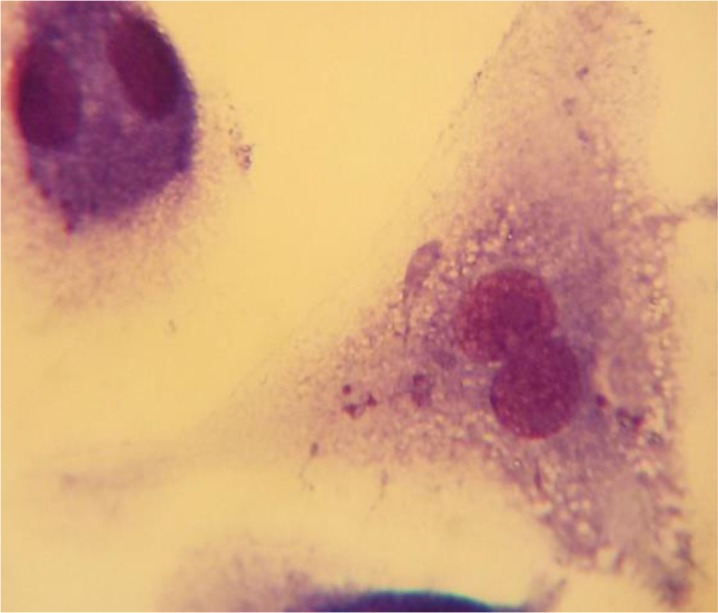
The effect of MA at a concentration of 12.63 ug/ml plus non-cytotoxic concentration of LLO (0.006 ug ml-1) on amastigote forms of L. major after 48 h. Incubation at 37°C, 5% CO2.

The results of the mean number of amastigotes in each macrophage showed that MA plus non-cytotoxic concentration of LLO (0.006 ug ml^−1^), combination efficiently reduced (*P*<0.05) the number of amastigotes forms of *L. major* in each macrophage in all concentrations when compared with MA or LLO alone. (Fig. 4). According to the calculation made by the statistical Sigma plot_13_ software the concentration of MA plus LLO, necessary to reduce by 50% the survival index of intracellular amastigotes was 12.63 μg ml^−1^ ± 0.13 that it indicated more effective anti-leishmanial effects on amastigote forms of *L. major* when compared with MA (IC50=46.17 μg ml^−1^±0.28) alone. In addition, results demonstrated that LLO (IC50= 1.72 μg ml^−1^ ± 0.07) alone induced more anti-amastigote effect than MA alone. Infected macrophages with no drugs (positive control) is shown in Fig. 5 and the amastigote suppressive effect of MA plus LLO at high concentration of MA after 48 h incubation at 37 °C, 5% CO2 is shown in Fig. 6. Moreover, the effect of MA at a concentration of 12.63 ug ml^−1^ alone, and MA at a concentration of 12.63 ug ml^−1^ plus 0.006 ug ml^−1^ LLO is shown in Fig. 7 and Fig. 8 respectively.

## Discussion

Leishmaniasis continues to be an important public health problem in endemic countries and WHO has put an ardent appeal for development of drugs and delivery strategies against it (24). The pentavalent antimony (Sb) such as meglumine antimoniate (MA) and sodium stibogluconate (Pentostam) are the standard recommended treatment for CL, but the major challenge in the treatment of leishmaniasis is the fact that the parasite lives inside resident macrophages, therefore therapeutic drugs face difficulties to penetrate inside the macrophages to kill the parasite (25).

Drug delivery systems are one of the most efficient ways of drug application that allows the adverse effects caused by problematic routes of administration to be avoided as well as increase the efficacy of the targeted material and to reduce the relevant toxicity (14). Carrier technology suggests an intelligent approach for drug delivery by joining the anti-leishmanial agents to a carrier particle such as polymers, nanoparticles, liposomes, nano-spheres, etc. (25). A polymer–drug (N-(2-hydroxypropyl) methacrylamide–amphotericin B (HPMA–AmB)) had a high antileishmanial in vitro and in vivo activity (26). In vitro antileishmanial activity of liposomes with different deformability properties and loaded with the photosensitizer zinc phthalocyanine (ZnP-cAL), was reported (27). Recently, carbohydrate (mannan, MN) functionalized PLGA (polylactide-co-glycolide) nanosphere was prepared in the treatment of murine VL. AmB-containing MN-PLGA nanocarriers can have potential application in the treatment of VL infection (28).

The potential use of pore-forming peptides to generate a novel class of anti-leishmanial drugs has not been explored yet. In this study, one pore-forming peptide, LLO, could induce cytotoxicity on murine macrophage cells (J774-A1) and amastigote form of *L. major*. The IC50 of LLO (48 h) was 1.72 μg ml^−1^ ± 0.07 against intracellular amastigotes. The LLO also showed a cytotoxic effect against the macrophage strain J774-A1. The value of 50% cytotoxic concentration was 2.56 μg ml^−1^ ± 0.09 showing that LLO is less toxic to macrophages than to the parasite. Moreover, noncytotoxic concentration of LLO for J774-A1 (0.006 ug ml^−1^), was able to much enhance the cytotoxicity induced by low dose of anti-leishmanial agent (MA) on amastigote form of *L. major*. We showed that various concentrations of MA along with non-cytotoxic concentration of LLO have higher anti-leishmanial effects against amastigote form of *L. major* (IC50=12.63 μg ml^−1^ ± 0.13) as compared with MA alone (IC50= 46.17 μg ml^−1^ ± 0.28). The overall mean IC50 values of MA plus noncytotoxic concentration of LLO for amastigote stage was significantly lower than those with MA alone (P<0.05).

The mechanism of potentiating amastigote death can be a direct consequence of increased drug delivery to the cytosol. LLO pores were estimated to be around 14 nm in diameter (19) and permit to passive diffusion of molecules at least ~45 kDa (29). Nearly all agents used to treat leishmaniasis have a molecular mass less than 45 kDa, including MA drug used in this study (364 Da) (30).

Generally, biological protein pores and pore-forming peptides such as LLO can create nanoscopic pathways for the flux of ions and other charged or polar molecules across cellular membrane. Biological pores are attractive for applications in nanomedicine such as cancer treatment, antimicrobial drug development, and drug delivery (31). LLO was explored as a delivery vehicle for macromolecules such as proteins, antigens or therapeutic molecules into cells both in vitro and in vivo (32). These LLO liposomes were employed as an efficient vaccine delivery system (33). Enhancement of the gene delivery efficiency with used of LLO in vitro using cultured cells (18). Moreover, pore-dependent membrane damage induced by pore-forming proteins is reversible (34).

## Conclusion

The combination of pore-forming proteins with anti-leishmanial agents could increase the outcome of anti-leishmanial drugs. These results open new prospects for research that can contribute to the development of products based on compounds from pore-forming proteins for the treatment of CL.
